# Mental health stigma in Ukraine: cross-sectional survey

**DOI:** 10.1017/gmh.2021.9

**Published:** 2021-03-22

**Authors:** Eleanor Quirke, Vitalii Klymchuk, Orest Suvalo, Ioannis Bakolis, Graham Thornicroft

**Affiliations:** 1 Hamburg University of Applied Sciences, Hamburg, Germany; 2 Institute for Social and Political Psychology of National Academy of Educational Sciences, Kyiv, Ukraine; 3 Institute of Mental Health of Ukrainian Catholic University, Lviv, Ukraine; 4 Department of Biostatistics and Health Informatics & Health Services and Population Research Department, London, UK; 5 Centre for Global Mental Health and Centre for Implementation Science, King's College London, Institute of Psychiatry, Psychology and Neuroscience, London, UK

**Keywords:** Discrimination, mental health, stigma, stigma intervention

## Abstract

**Background and study objectives:**

This study aimed to assess among Ukrainian adults: (1) knowledge of mental disorders; (2) attitudes towards people with mental health disorders, and to the delivery of mental health treatment within the community; and (3) behaviours towards people with mental disorders.

**Methodology:**

A cross-sectional survey of Ukrainian adults aged 18–60 was conducted. Stigma-related mental health knowledge was measured using the mental health knowledge schedule. Attitude towards people with mental health disorders was assessed using the Community Attitudes towards Mental Illness scale. The Reported and Intended Behaviour scale was used to assess past and future intended behaviour towards people with mental health disorders.

**Results:**

Associations between gender, age, and educational level and the knowledge and attitudes measures were identified. There was evidence of a positive association between being male and positive intended behaviours towards people with mental health disorders [mean difference (MD) = 0.509, 95% confidence interval (CI) 0.021–0.998]. Older age was negatively associated with positive intended behaviours towards people with mental health disorders (MD = −0.017, 95% CI 0.0733 to −0.001). Higher education was positively associated with stigma-related mental health knowledge (MD = 0.438, 95% CI 0.090–0.786), and negatively associated with authoritarian (MD = 0.755, 95% CI 0.295–1.215) attitudes towards people with mental health problems.

**Conclusion:**

Overall, the findings indicate a degree of awareness of, and compassion towards, people with mental illness among Ukrainian adults, although this differed according to gender, region, and education level. Results indicate a need for the adoption and scaling-up of anti-stigma interventions that have been demonstrated to be effective.

## Introduction

Stigma refers to the process by which characteristics are assigned to a person or phenomena, leading to the negative perception and discrediting of an individual or phenomenon (Goffman, 2009). In mental health, stigma is understood as comprising three elements, namely problems of knowledge (ignorance), problems of attitude (prejudice), and problems of behaviour (discrimination) (Thornicroft *et al*., 2007).

The implications of mental health stigma are significant and wide-ranging. Stigma of mental health disorders reduces the opportunities of those living with mental health disorders through unemployment, loss of income, as well as reduced access to housing and education (World Health Organization, 2002; WHO, 2005; Thornicroft, 2006; Patel *et al*., 2010). Mental health stigma also impacts the health and wellbeing of those living with mental health disorders, thereby reducing their quality of life (Winkler *et al*., 2015). For example, people with mental health disorders often receive differential treatment for physical health conditions, contributing to the higher morbidity and premature mortality of those living with mental health disorders (Thornicroft, 2011, 2013). Mental health stigma is further associated with lower participation in healthcare, with negative experiences in healthcare settings leading those with mental health disorders to delay or avoid seeking treatment (Henderson *et al*., 2013; Clement *et al*., 2015).

Knowledge of public understanding and attitudes towards mental health can serve as the starting point for any intervention program seeking to reduce mental health stigma and enhance engagement with mental health services. However, knowledge related to public mental health stigma is mostly limited to wealthier countries, with the vast majority of research conducted in North-Western Europe (Evans-Lacko *et al*., 2014). In particular, there is limited evidence on mental health stigma in Central and Eastern European Countries (Evans-Lacko *et al*., 2014). The few studies that have been conducted indicate high levels of stigma (Winkler *et al*., 2015, 2016; Buchman-Wildbaum *et al*., 2018). Nevertheless, given the paucity of studies on the topic of mental health stigma in this region, it is difficult to say whether these levels of stigma are higher, or qualitatively different, than other countries in Western Europe (Winkler *et al*., 2016). In view of this, a call for further study into public mental health stigma in new market economies in Europe has been made (Buchman-Wildbaum *et al*., 2018).

Against this background, the aim of the present study was to examine public knowledge of mental disorders, as well as public attitudes and intended behaviour towards people with mental disorders within Ukraine. The prevalence of mental disorders in Ukraine is high, with one in three Ukrainians experiencing at least one mental health disorder in their lifetime (Bromet *et al*., 2005). The most common mental disorders in Ukraine include alcohol disorders, mood disorders, and anxiety disorders (Bromet *et al*., 2005; Tintle *et al*., 2011), with overall prevalence for disorders such as depression found to be substantially higher in Ukraine compared to Western European countries (Tintle *et al*., 2011). Occupation, damaged infrastructure, and displacement as a result of the ongoing war in the Eastern region of the country have resulted in higher rates of mental health disorders in the East as well as among internally displaced persons in Ukraine (Colborne, 2015; Kuznestsova *et al*., 2019; Roberts *et al*., 2019). An institution-based health system that emphasizes a biomedical and exclusionary approach on mental health treatment has been identified as a key factor for the large treatment gap for mental health disorders seen in Ukraine (Weissbecker *et al*., 2017).

The Ukrainian government has acknowledged the need to reform the mental health system in order to meet the needs of the population (Ministry of Health of Ukraine, 2017). Mental health reform comprises a key part of overall health system reform in Ukraine, which has already seen the successful implementation of a number of structural changes, namely the institution of a single purchaser of health services, as well as reform in the financing of the primary care sector (Ministry of Health of Ukraine, 2017). In its 2017 Mental Health Concept Note, the Ministry of Health identified a number of issues within the mental health system, including the lack of human rights protection in mental health legislation, inadequate regulation of the mental health sector, as well as too great a focus on the delivery of mental health services in specialized and/or institutionalized settings (Ministry of Health of Ukraine, 2017). Poor accessibility of services due to stigma, a lack of awareness of mental health issues in the general population, high levels of stigma towards mental disorders, as well as people with mental disorders, were also identified as key issues. A range of measures were proposed by the concept note to tackle these issues, including greater mental health service delivery in the community, the integration of mental health services into primary care, as well as raising awareness around mental health and reducing stigma (Ministry of Health of Ukraine, 2017).

Current evidence on stigma towards mental health within the Ukrainian population is limited. One study conducted in 2008 by the Kyiv International Institute of Sociology (KIIS) aimed to explore feelings toward people with mental health conditions. Among the analysed sample (*N* = 2046), compassion towards people with mental health disorders was most frequently reported (84.7% men and 82.2% women), followed by fear (5.9% men and 13.2% women). In total, 20.0% of respondents identified aggression as a trait of individuals with a mental disorder (Kostyuchenko *et al*., 2008).

A more recent study on the mental health of individuals living in the conflict-affected regions in the East of Ukraine (*n* = 1030) also examined attitudes towards mental health disorders, as well as examined treatment-seeking behaviours. In total, 37.1% of respondents consider attending a specialist as a sign of weakness, whereas 33.5% of respondents consider it better to avoid people with disorders, so as to avoid getting such a problem themselves. Although 41.1% of respondents reported that they would tell someone close to them if they had a disorder themselves, 16.3% reported that they would keep their disorder to themselves (Kyiv International Institute of Sociology, 2018). Furthermore, a small study conducted in one region of Central Ukraine (700 respondent, 2018) found that while respondents are accepting of people with mental disorders as neighbours and users of common services, respondents exhibited high levels of social distance, not wanting people with mental disorders as friends or work colleagues (Savychenko and Portnytska, 2018).

This study sought to build on current evidence on mental health stigma in Ukraine by conducting a cross-sectional study using a population-based sample of Ukrainian adults. Specifically, this study aimed to assess: (1) knowledge of mental disorders and treatment of mental health disorders; (2) attitudes towards people with mental health disorders, and towards the delivery of mental health treatment within the community; and (3) behaviours towards individuals with mental disorders. This survey can act as a baseline for future surveys to monitor national trends.

## Methods

A cross-sectional survey of adults aged 18–60 years was conducted. This survey was conducted online using computer-assisted web interviewing in January 2020 by InMind, Ukraine-based health and research consulting company. Participants were recruited using an existing online access panel managed by InMind. This panel covers both rural and urban regions across Ukraine and covers a range of educational levels and socioeconomic groups. Panel members were recruited via targeted face to face recruiting. Panel members were also recruited via internet-based advertising. The upper age limit of 60 was chosen in view of the online format of the survey. A random selection of the existing survey panel was conducted to obtain the current sample.

All respondents were required to give their consent prior to completing the survey. In line with Ukrainian requirements, ethical approval for the survey was not required as thresholds for ethical approval were not met.

### Survey items

A range of measures were employed by the survey.

#### Knowledge and awareness of mental health disorders

A range of questions were used to assess respondents' understanding and conceptualization of mental health disorders, including; Can you tell if someone has a mental problem? If yes, what are the signs and symptoms of them? In addition, the mental health knowledge of the respondents was assessed using the Mental Health Knowledge Schedule (MAKS) (Evans-Lacko *et al*., 2010). The schedule comprises a total of 12 items. The first six items assess stigma-related mental health knowledge, namely help seeking, recognition, support, employment, treatment, and recovery. The remaining six items assess knowledge about specific mental health conditions. Respondents indicate their agreement with each of the items using a Likert scale (i.e. where 1 = strongly disagree and 5 = strongly agree). A total score was calculated across the first six items, with a higher total score indicating greater knowledge (total score range, 5–30). Cronbach's *α* in this study was 0.28, which is lower than in other studies (see for example Evans-Lacko *et al*., 2010). However, given the MAKS was not primarily developed to function as a scale (Evans-Lacko *et al*., 2010), and the response to the individual items provide useful insights, the scale was retained. This is discussed in the strengths and weaknesses section of the article.

#### Behaviour towards people with mental illness, or in response to mental disorders

The Reported and Intended Behaviour scale (RIBS) was used to assess past and intended future behaviour towards people with mental health disorders. The scale comprises two subscales, the first of which assess past and present interactions with people with experience of mental disorders. The second subscale assesses how individuals intend to interact with people with experiences of mental disorders in the future (or, how willing the respondent is to accept a person with mental problems). Respondents indicate their agreement with the eight items of the scale using a Likert scale (i.e. where 1 = strongly disagree and 5 = strongly agree). In this study, a total score across the 4 items related to intended behaviour was calculated, where a higher total score indicates more positive intended behaviour towards those with mental illness (total score range = 4–20). Cronbach's *α* in this study was 0.87.

#### Attitudes towards people with mental health disorders

Attitudes towards people with mental health disorders were assessed using the Community Attitudes towards Mental Illness (CAMI) scale (Taylor and Dear, 1981). The CAMI assesses attitudes towards people with mental disorders living in the community and comprises four subscales, Authoritarian, Social Restrictiveness, Benevolence and Community Mental Health Ideology. Each subscale comprises 10 items, and respondents indicate their agreement with each of the items using a Likert scale (i.e. where 1 = strongly agree and 5 = strongly disagree). The Authoritarian subscale includes statements related to the need to hospitalize the mentally ill, differences between those living with a mental illness and those living without, the importance of custodial care and the causes of mental illness. A higher total Authoritarian subscale score indicates a less authoritarian attitude towards individuals with mental illness (total score range, 5–50). The Benevolence subscale includes items related to the need to be sympathetic towards those with mental illness and the responsibility society has to care for the mentally ill. A higher total Benevolence subscale score indicates a less benevolent attitude towards individuals with mental illness (total score range, 5–50). The Social Restrictiveness subscale measures the degree to which individuals find those living with mental disorders threatening and dangerous. A higher total Social Restrictiveness subscale score indicates less restrictive attitudes towards individuals with mental illness (total score range, 5–50). The Community Mental Health Ideology (CMHI) subscale assesses attitudes towards caring for those with mental health disorders in the community. A higher total score for this subscale indicates a less positive attitude towards delivering mental health services in the community (total score range, 5–50). Cronbach's *α* in this study was 0.46 for the Authoritarian subscale, 0.70 for the Benevolence subscale, 0.67 for the Social Restrictiveness subscale, and 0.78 for the CMHI subscale.

### Statistical analysis

Following data collection, representativeness of the sample in terms of gender, age, and region of residence was ensured via calibration weighting. Representativeness of the data across these three key variables was defined according to official data from the State Statistics Services of Ukraine (collected in 2019). Sample characteristics of the analytical sample were then calculated.

Descriptive analysis was conducted (Table 1). Comparisons of responses across sex (male and female) and age groups (18–30, 31–40, 41–50, and 51–60) were conducted. In addition, the mean total scores for the MAKS, RIBS, and CAMI were computed.

**Table 1.** Descriptive characteristics of the sample

	*N*/Mean	%/s.d.
Gender (female)	514	51%
Age	39.2	10.95
Income	10.01	24.62
Region		
North-Center	342	34%
West	282	28%
East	262	26%
South	121	12%
Education		
Higher education	547	54%
Other	460	46%

*Notes*: Household income measured using the KIIS subjective assessment of the household financial situation. North-Center comprises Kyiv, Sumy, Vinnytsa, Zhytomyr, Kirovohrad, Chernihiv, Poltava, and Cherkasy oblasts; West comprises Lviv, Ivano-Frankivsk, Khmelnytsky Rivne, Ternopil, Zakarpatie, Volyn, and Chernivtsi oblasts; East comprises Dnipropetrovsk, Kharkiv, Donetsk and Zaporizhzhya, and Luhansk oblasts; South comprises Odesa, Mykolaiv, and Kherson oblasts. Higher education comprises those who have completed tertiary education (bachelor, master, or doctorate); others are comprised of those who have not completed secondary school, completed secondary school, completed technical school, or have not yet completed tertiary education.

Potential associations between gender, age, place of residence and education level, and the total scores of MAKS, RIBS, and CAMI subscales were explored with the use of linear regression models.

IBM SPSS Statistics 26 software was used.

## Results

### Sample demographics

A total of 1007 individuals participated in the online survey. The response rate for this survey, calculated by dividing the sum of all those who completed the survey by the sum of those invited to complete the survey, was 32%. Sample demographics can be found in Table 1.

### Knowledge and awareness of mental health disorders

The findings indicate limited knowledge and understanding of mental health disorders. The majority (61%) of respondents indicated they were unsure they would be able to determine if someone has a mental health disorder. One-fifth (21%) of respondents who indicated they could determine if someone has mental health disorders identified inadequate behaviour, aggression, speech disorders, and inappropriate facial expressions or gestures as defining signs and symptoms of a mental health disorder. In terms of knowledge about specific mental health disorders, 91% of respondents agreed that schizophrenia is a type of mental illness, 63% of respondents agreed that drug addiction was a mental illness, and 45% of respondents consider depression to be a type of mental illness (see Appendix 1 for further detail). The mean total MAKS score was 19.52 (s.d.: 2.84, range: 9–28), underscoring the limited understanding of mental health disorders among the population (see Table 2).

**Table 2.** Mean and median total scores for MAKS, RIBS, and CAMI subscales

	Mean (s.d.)	Median; range (IQR)
MAKS	19.52 (2.83)	19; 9–28 (3)
RIBS	10.63 (3.96)	11; 4–20 (5)
CAMI subscales		
Authoritarian	31.67 (3.66)	32; 21–46 (5)
Benevolent	23.77 (4.23)	24; 10–40 (6)
Social restrictiveness	29.79 (4.48)	30; 14–46 (6)
CMHI	30.32 (5.06)	30; 10–50 (6)

*Notes*: Response options for the MAKS and RIBS included strongly agree, agree, neutral, disagree, strongly disagree, and do not know. Do not know responses recoded to neutral to calculate MAKS total score.

### Behaviours towards people with mental illness or in response to mental health disorders

The most common interaction respondents have with mental illness is through having or having had a neighbour with a mental health problem (39%). Just under one-fifth (19%) of respondents have, or have had, worked with someone with a mental health problem, and 20% of respondents have, or have had, a close friend with a mental health problem (see Appendix 1 for further details). The mean total RIBS score was 10.63 (s.d. = 3.96, range: 4–20), indicating limited positive intended behaviour towards people with mental health problems (or otherwise described as a greater social distance from those with mental illness).

### Attitudes towards people with mental health disorders

Overall, respondents demonstrated a high degree of empathy towards people with mental illness. The majority (84%) of respondents disagreed that the mentally ill do not deserve sympathy, and 73% of respondents agreed that a more tolerant attitude towards people with mental disorders should be adopted within society. Nevertheless, responses to the CAMI indicated stigmatizing attitudes towards those with mental disorders. For example, 79% of respondents somewhat or strongly agreed with the statement that anyone with a history of mental problems should be excluded from taking public office. In terms of treatment of mental disorders, 86% of individuals either somewhat or strongly believed that mental health patients need the same kind of control and discipline as a young child. Moreover, 46% believed that as soon as a person shows signs of mental disturbance, they should be hospitalized. However, 75% of respondents agreed that psychiatric hospitals seem more like prisons than places where the mentally ill can be cared for, and 83% of respondents either somewhat or strongly agreed with the statement that we have a responsibility to provide the best possible care for the mentally ill (see Appendix 1 for further detail).

The mean total scores of the Authoritarian and Social Restrictiveness reflect the high degree of empathy but stigmatized attitudes towards people with individuals with mental illness (see Table 2). The total mean score of the Authoritarian subscale was 31.67 (s.d. = 3.66, range: 21–46), whereas the total mean score of the Social Restrictiveness subscale was 29.79 (s.d. = 4.48, range: 14–46). The total mean scores of the Benevolence and CMHI subscales were 23.77 (s.d. = 4.23, range: 10–40) and 30.32 (s.d. = 5.06, range: 10–50), respectively.

### Associations of the MAKS, RIBS, and CAMI subscales with sociodemographic variables

The results of multiple linear regression are depicted in Table 3. There was evidence of a positive association between being male and positive intended behaviours towards people with mental health disorders [mean difference (MD) = 0.509, 95% confidence interval (CI) 0.021–0.998]. We also observed a negative association between gender (male *v*. female) and benevolent attitudes towards people with mental health disorders (MD = 1.077, 95% CI 0.559–1.596), with less benevolent attitudes among men compared with women.

**Table 3.** Associations between sociodemographic characteristics and total scores of MAKS, RIBS scales and CAMI subscales

	MAKS	RIBS	CAMI subscales			
			Authoritarian	Benevolence	Social Restrictiveness	Community Mental Health Ideology
Gender (reference: female)	−0.292 (−0.637 to 0.052)	0.509* (0.021–0.998)	−0.427 (−0.882 to 0.028)	1.077*** (0.559–1.596)	0.116 (−0.425 to 0.657)	0.227 (−0.399 to 0.853)
Age in years	−0.017* (−0.033 to −0.001)	−0.051*** (−0.073 to −0.28)	−0.002 (−0.023 to 0.019)	0.011 (−0.013 to 0.035)	−0.074*** (−0.099 to −0.050)	0.033* (0.005–0.062)
Income	−0.001 (−0.008 to 0.005)	−0.003 (−0.012 to 0.007)	−0.001 (−0.010 to 0.008)	0.008 (−0.002 to 0.018)	−0.003 (−0.014 to 0.007)	0.010 (−0.003 to 0.023)
Region (reference: West)						
North-Center	−0.475* (−0.899 to −0.051)	−0.032 (−0.633 to 0.568)	−0.688* (−1.284 to −0.128)	0.984** (0.346–1.621)	−0.881** (−1.546 to −0.216)	1.006* (0.237–1.776)
East	0.054 (−0.429 to 0.537)	−0.098 (−0.783 to 0.587)	−0.508 (−1.147 to 0.130)	0.647 (−0.081 to 1.374)	−0.609 (−1.368 to 0.149)	0.303 (−0.574 to 1.181)
South	0.011 (−0.563 to 0.584)	0.204 (−0.608 to 1.017)	−1.056** (−1.814 to −0.299)	−0.368 (−1.231 to 0.495)	−0.519 (−1.418 to 0.381)	0.007 (−1.034 to 1.048)
Education (reference: Other)	0.438* (0.090–0.786)	0.472 (−0.021 to 0.965)	0.755 **(0.295–1.215)	−0.891** (−1.415 to −0.368)	0.349 (−0.197 to 0.895)	0.057 (−0.575 to 0.689)
Constant	20.283*** (19.548–21.017)	12.223*** (11.182–13.264)	32.057*** (31.087–33.028)	22.708*** (21.602–23.813)	33.079*** (31.926–34.232)	28.328*** (26.994–29.662)

Mean difference and 95% confidence intervals (CIs) are presented.

*Notes*: ***p *<* 0.001, **p *<* 0.01, *p *<* 0.05. Beta-coefficients are reported; 95% CI in parentheses. Household income measured using the KIIS subjective assessment of household financial situation. North-Center comprises Kyiv, Sumy, Vinnytsa, Zhytomyr, Kirovohrad, Chernihiv,Poltava, and Cherkasy oblasts; West comprises Lviv, Ivano-Frankivsk, Khmelnytsky Rivne, Ternopil, Zakarpatie, Volyn, and Chernivtsi oblasts; East comprises Dnipropetrovsk, Kharkiv, Donetsk and Zaporizhzhya, and Luhansk oblasts; South comprises Odesa, Mykolaiv, Kherson oblasts. Higher education comprises those who have completed tertiary education (bachelor, master or doctorate); others are comprised of those who have not completed secondary school, completed secondary school, completed technical school or have not yet completed tertiary education.

Older age was negatively associated with positive intended behaviours towards people with mental health disorders (MD = −0.017, 95% CI −0.0733 to −0.001). Older age was also negatively associated with less socially restrictive attitudes (MD = −0.074, 95% CI −0.099 to −0.050). Furthermore, older age was associated with less positive attitudes towards delivering mental health services in the community (MD = 0.033, 95% CI 0.005–0.062).

Lesser stigma-related mental health knowledge was found among respondents living in the Northern and Central region of Ukraine (MD = −0.475, 95% CI 0.899 to −0.051) compared to those living in the Western region of Ukraine. We also found that residents living in the Northern and Central region of Ukraine (compared to the western region) had more authoritarian (MD = −0.688, 95% CI −1.284 to −0.128), more socially restrictive attitudes (MD = −0.881, 95% CI −1.546 to −0.216), less benevolent attitudes (MD = 0.984, 95% CI 0.346–1.621) and lesser acceptance of caring for people with mental health problems in the community (MD = 1.006, 95% CI 0.237–1.776).

Higher education was positively associated with stigma-related mental health knowledge (MD = 0.438, 95% CI 0.090–0.786) and negatively associated with authoritarian attitudes (MD = 0.755, 95% CI 0.295–1.215) towards people with mental health problems.

There was no evidence of an association between household income and the three knowledge and attitude measures.

## Discussion

The findings of this survey indicate a degree of awareness of, concern for, and compassion towards, people with mental illness. Nevertheless, overall, there is a high lack of knowledge and understanding about most types of mental illness among adults in Ukraine, and in particular a limited understanding of the efficacy of treatment for mental health disorders. Respondents showed reasonably high levels of empathy for those with mental disorders and overwhelmingly agreed that mental healthcare should be not only of high quality but also be improved from what it is currently. However, this did not mean that respondents agree that mental health services should be delivered in the community, with the majority of respondents finding that such services would downgrade a neighbourhood and present a security risk.

Our findings align with studies conducted in comparable countries, namely in Hungary and Czech Republic high levels of social distance and rejection of individuals with mental disorders were reported (Winkler *et al*., 2015, 2016; Buchman-Wildbaum *et al*., 2018). Our study differs slightly from those conducted in Hungary and Czech Republic with respect to gender differences, namely the three studies identified more accepting behaviours towards those with mental health problems among women (Winkler *et al*., 2015, 2016; Buchman-Wildbaum *et al*., 2018). Buchman-Wildbaum *et al.* (2018) similarly reported an association between education level and social distance. Although the present study found associations between age and knowledge of, and attitudes towards, mental health disorders, the studies conducted in Hungary and Czech Republic reported mixed findings (Winkler *et al*., 2015, 2016; Buchman-Wildbaum *et al*., 2018).

The high levels of social distance and rejection of individuals with mental disorders found in Hungary and Czech Republic have been attributed in part to communist rule in this region. During the era of communism, social problems were considered consequences of capitalism or the result of anti-government activities, and mental illnesses were considered individual problems and unrelated to society (Winkler *et al*., 2015, 2016; Buchman-Wildbaum *et al*., 2018). Those with mental illnesses were socially excluded and hospitalized in large psychiatric asylums away from the community (Winkler *et al*., 2015, 2016; Buchman-Wildbaum *et al*., 2018). The high levels of social distance and rejection identified in the studies have therefore been characterized as a hangover effect of communism in these countries; with such characterizations of mental illness, as well as the treatment of those with mental illness, remaining present in the public's minds (Winkler *et al*., 2015; Buchman-Wildbaum *et al*., 2018).

Ukraine shares this history of communist rule. Reviews of mental health services in Ukraine have noted the lasting impact of Soviet conceptualizations and treatment of mental health disorders on public understandings of mental health, as well as the willingness of the public to engage with the mental health system (Weissbecker *et al*., 2017). The ongoing institutionalization of people with mental disorders in Ukraine, and contemporary examples of violation of the human rights of individuals with mental disorders (Keukens, 2017; Weissbecker *et al*., 2017; Council of Europe, 2018), will not serve to alter these perceptions of the public. Indeed, Weissbecker *et al*. (2017) noted in their comprehensive review of the Ukrainian mental health system, that people with mental illness seek to avoid engaging with mental health services due to unpleasant experiences in the past, as well as the fear that seeking help results in the restriction of their rights.

The findings of our study therefore emphasize the need in Ukraine to undertake action. Stigma is not immovable, and despite its challenging history, Ukraine is able to tackle the misunderstandings and stigmatization of mental illness and those who have mental disorders. A range of systematic reviews have demonstrated that stigma interventions can reduce stigmatizing attitudes and discrimination, and improve mental health knowledge (See for example, Corrigan *et al*., 2012; Borschmann *et al*., 2014; Thornicroft *et al*., 2016; Gronholm *et al*., 2017; Morgan *et al*., 2018). Decades of experience in anti-stigma programmes has shown that any country, irrespective of its size, wealth or level of development demonstrates that successful programmes against stigma can be implemented (Sartorius, 2006). Doing so not only has benefits for those with mental disorders, and those that are close to them. It also has wide-ranging benefits for society (Callard *et al*., 2008).

There are some excellent recent examples of comprehensive anti-stigma campaigns that have been implemented in recent years. One well-known example is the Time to Change campaign, the current national programme against stigma and discrimination in England. Funded by the national government, the campaign comprises actions at the national and local level and engages individuals, communities, and stakeholders. Time to Change has conducted mass-media marketing campaigns targeted towards the adult population and has used social media, such as Facebook and Twitter, to deliver key messages. At the local level, anti-discrimination initiatives have been implemented, as have exercise programmes for people with mental disorders to promote social contact. A range of educational programs have also been implemented to educate, change attitudes, and reduce discrimination among medical students, trainee teachers, and employers. A key component of the campaign has also been to improve the reporting and portrayal of mental illness in journalism and other media. The campaign has consistently communicated that mental illnesses are common and that people with mental disorders can lead meaningful lives. The campaign has positioned mental illness as the ‘last taboo,’ and has emphasized that everyone can do something to help people with mental disorders (Henderson and Thornicroft, 2009).

The Time to Change campaign in England has been successful. An evaluation of the programme's activities from 2007–2017 found significant improvements in all mental health stigma-related outcomes in England (Robinson and Henderson, 2019). This evaluation confirmed the findings of other evaluations of the programme, including improvements in knowledge of, and attitudes towards, mental illness, an increase in contact between people with mental health disorders, and decreases in the desire for social distance (Henderson *et al*., 2016). An evaluation of the programme has also found evidence for a reduction in discrimination against mental health service users (Corker *et al*., 2016).

The Time to Change campaign has been supported by a multi-million budget, something that may not be as available to Ukraine. Nevertheless, there are a number of aspects of the Time to Change campaign, as well as other successful interventions, that should be considered when devising a campaign for the Ukrainian context.

First, all programmes should seek to involve people with experience with mental disorders, and those close to them, in the planning and implementation of anti-stigma campaigns. Anti-stigma campaigns must deal with problems experienced by those who are stigmatized, and such individuals are the subject matter experts in the experience of stigma, and what change is most important for their situation to be improved (Sartorius, 2006). In line with this, interventions that seek to address stigma and discrimination must empower these individuals and give them a voice. This can be through the day-to-day functions of the programme, or through initiatives, such as a speakers' bureaux that trains people with experience in mental disorders to talk with the media and other organizations (Callard *et al*., 2008).

Second, the key ingredient to all anti-stigma campaigns is social contact. Social contact refers to interactions of individuals without mental health problems, with people with experience of mental health problems. Social contact challenges previous perceptions and stereotypes of people with mental health problems, thereby increasing empathy, reducing anxiety, and leading to behaviour change among those without the experience of mental health disorders (Couture and Penn, 2003). Interventions that include variants of social contact have been found to be the most effective in reducing public mental health stigma (Corrigan *et al*., 2012; Thornicroft *et al*., 2016). Live social contact has been found to be more effective than filmed contact (Corrigan *et al*., 2012). Furthermore, social contact that ensures equal status between groups or participants, that involves inter-group cooperation, as well as common goals for the interaction, has been found to be most effective (Thornicroft *et al*., 2016).

Third, anti-stigma programmes should clearly define the audiences that it is seeking to reach and tailor its messages for these target groups. A number of anti-stigma campaigns target specific target groups (i.e. the media or young people), in addition to mass-media campaigns. This has led to campaigns operating not only at the national level but also at the local level, where interventions can be more closely tailored to the target audience (Sartorius, 2006).

In line with this, it is key that anti-stigma programmes are sensitive to the cultural context in which they are implemented. Although stigma is a universal experience, there are culturally and regionally specific experiences of stigma (Koschorke *et al*., 2017). The planning of anti-stigma programmes thus needs to consider the understanding and acceptance of mental illnesses within target audiences, as well as how stigma manifests itself in the audience's cultural and social context.

The messaging of the campaign should also be clearly defined, tailored to the target audience and approach the topic of mental illness in a way that has been proven to be effective. A consensus study conducted with experts in the field of mental health stigma recommended messages that are recovery oriented and emphasize the personhood of individuals with mental disorders (Clement *et al*., 2010). This study also discouraged messages that include biological causal explanations (Clement *et al*., 2010). Recent evidence supports that these messages are best avoided (Schomerus *et al*., 2012). First-person narratives, as well as social inclusion or human rights messages, were identified in a Cochrane review of mass-media interventions to be most effective in reducing prejudice (Clement *et al*., 2013).

Sixth, anti-stigma intervention should be implemented over the long-term to ensure a sustainable impact. Although interventions have been demonstrated to reduce stigma and discrimination over the short-term, there is no evidence to suggest that interventions can ensure reduced stigma over time (Morgan *et al*., 2018). Interventions should therefore be implemented on an ongoing basis to ensure that progress is not squandered.

Finally, it is key for anti-stigma programmes to be evaluated and the findings of these evaluations to be reported. These evaluations should assess the programme in terms of (1) improvements made in target areas identified by those with experience in mental illness, as well as (2) the behaviour of individuals towards people with mental disorders, and those living with mentally disordered people. This is particularly pertinent to any interventions undertaken in Ukraine, given the dearth of literature on stigma interventions in low- and middle-income countries, and in Central and Eastern European countries.

Our study has a number of strengths. First, our study used established measures for measuring knowledge and awareness of mental health disorders, as well as attitudes and behaviours towards people with mental health disorders, providing a comprehensive understanding of public stigma towards mental health among Ukrainian adults. There is currently very limited knowledge about mental health stigma in Ukraine and Eastern Europe, therefore this study provides unique insights. Furthermore, the online nature of the survey may have reduced desirability bias, an issue associated with surveys related to more sensitive topics such as mental health.

The study also has some limitations. Although the survey has a good sample size with reasonable statistical power, its online nature and age limitation up to 60 years old may have resulted in some selection bias. The survey measures used in the survey have not yet been validated in Ukraine. The survey questionnaire was created by English-speaking and Ukrainian-speaking mental health professionals, and the MAKS, RIBS, and CAMI were translated by Ukrainian-speaking experts. Nevertheless, findings may have been shaped by this translation approach, as well as by differences in the English and Ukrainian languages. The translation of the MAKS survey, for example, may account for its low Cronbach's *α* in this study. Findings related to the MAKS survey, in particular, should therefore be interpreted with caution.

## Conclusions

Overall, the findings of this survey indicate a degree of awareness of, concern for, and compassion towards, people with mental illness, however, this varies according to gender, age, and educational level. Nevertheless, overall, there is a high lack of knowledge and understanding about most types of mental illnesss among adults in Ukraine and a high degree of social distance. There is a clear need for anti-stigma interventions in Ukraine. We encourage the adoption and scaling-up of interventions that have been demonstrated to be effective, and in particular interventions that have social contact as the key ingredient.

## Appendix 1

**Table:**

Measure			%
Awareness and knowledge of mental health			
Can you tell if someone has a mental health problem? [yes, no, hard to say]		–	–
–		Yes	21
–		No	18
–		Hard to say	61
What are the signs and symptoms of a mental health problem? [open-ended]		–	–
–		Strange and inappropriate behaviour	39
–		Aggression	25
–		Speech disorders	16
–		Inappropriate facial expressions, gestures	11
–		Mood swings	11
–		Irritability	10
Mental health knowledge schedule (MAKS)			
Psychotherapy can be an effective treatment for people with mental health problems		–	–
–		Agree	57
Medication can be an effective treatment for people with mental health problems		–	–
–		Agree	50
Most people with mental health problems want to have paid employment		–	–
–		Agree	45
Most people with mental health problems go to a healthcare professional to get help		–	–
–		Agree	36
If a friend had a mental health problem, I know what advice to give them to get professional help		–	–
–		Agree	35
People with severe mental health problems can fully recover		–	–
–		Agree	22
Schizophrenia is a mental health disorder		–	–
–		Agree	91
Bipolar disorder is a mental health disorder		–	–
–		Agree	79
Drug addiction is a mental health disorder		–	–
–		Agree	63
Grief is a mental health disorder		–	–
–		Agree	22
Depression is a mental health disorder		–	–
–		Agree	45
Stress is a mental health disorder		–	–
–		Agree	27
*Reported and Intended Behaviour scale*			
Reported behaviour items			
Are you currently living with, or have ever lived with someone with a mental health problem?	–		–
–	Yes		19
Are you currently working with, or have ever worked with someone with a mental health problem?	–		–
–	Yes		19
Do you have, or have you ever had, a neighbour with a mental health problem?	–		–
–	Yes		39
Do you have, or have you ever had, a close friend with a mental health problem? (yes, no)	–		–
–	Yes		20
Intended behaviour items			
In the future, I would be willing to live with someone with a mental health problem	–		–
–	Agree		13
In the future, I would be willing to work with someone with a mental health problem	–		–
–	Agree		22
In the future, I would be willing to live nearby to someone with a mental health problem	–		–
–	Agree		23
In the future, I would be willing to continue a relationship with a friend who developed a mental health problem	–		–
–	Agree		38
CAMI – Authoritarian subscale			
Mental health patients need the same kind of control and discipline as a young child	–		–
–	Agree		86
As soon as a person shows signs of mental disturbance, he should be hospitalized	–		–
–	Agree		46
There is something about the mentally ill that makes it easy to tell them from normal people	–		–
–	Agree		40
One of the main causes of mental illness is a lack of self-discipline and will power	–		–
–	Agree		29
The best way to handle the mentally ill is to keep them behind locked doors	–		–
–	Agree		19
Less emphasis should be placed on protecting the public from the mentally ill	–		–
–	Disagree		48
Mental hospitals are an outdated means of treating the mentally ill	–		–
–	Disagree		24
Mental illness is an illness like any other	–		–
–	Disagree		18
The mentally ill should not be treated as outcasts of society	–		–
–	Disagree		7
Virtually anyone can become mentally ill	–		–
–	Disagree		5
CAMI – Benevolence subscale			
We have the responsibility to provide the best possible care for the mentally ill	–		–
–	Agree		83
Our mental hospitals seem more like prisons than like places where the mentally ill can be cared for	–		–
–	Agree		75
We need to adopt a far more tolerant attitude toward the mentally ill in our society	–		–
–	Agree		73
The mentally ill have for too long been the subject of ridicule	–		–
–	Agree		66
More tax money should be spent on the care and treatment of the mentally ill	–		–
–	Agree		66
The mentally ill do not deserve our sympathy	–		–
–	Disagree		84
Increased spending on mental health services is a waste of tax dollars	–		–
–	Disagree		74
There are sufficient existing services for the mentally ill	–		–
–	Disagree		54
The mentally ill are a burden on society	–		–
–	Disagree		49
It is best to avoid anyone who has mental problems	–		–
–	Disagree		20
CAMI – Social Restrictiveness subscale			
Anyone with a history of mental health problems should be excluded from taking public office	–		–
–	Agree		79
I would not want to live next door to someone who has been mentally ill	–		–
–	Agree		56
The mentally ill should be isolated from the rest of the community	–		–
–	Agree		32
A woman would be foolish to marry a man who suffered from mental illness, even if he seems fully recovered	–		–
–	Agree		31
The mentally ill should not be given any responsibility	–		–
–	Agree		15
Most women who were once patients in a mental hospital can be trusted as babysitters	–		–
–	Disagree		58
No one has the right to exclude the mentally ill from the neighbourhood	–		–
–	Disagree		31
The mentally ill are less of a danger than most people suppose	–		–
–	Disagree		24
The mentally ill should not be denied their individual rights	–		–
–	Disagree		15
Mental patients should be encouraged to assume the responsibilities of normal life	–		–
–	Disagree		9
CAMI – Community Mental Health Ideology (CMHI)			
As far as possible mental health services should be provided through community-based facilities	–		–
–	Agree		66
The best therapy for many mental health patients is to be part of a normal community	–		–
–	Agree		54
Residents have nothing to fear from people coming into their neighbourhood to obtain mental health services	–		–
–	Agree		42
Residents should accept the location of mental health facilities in their neighbourhood to serve the needs of the local community	–		–
–	Agree		41
Locating mental health services in residential neighbourhoods does not endanger local residents	–		–
–	Agree		32
Local residents have good reason to resist the location of mental health services in their neighbourhood	–		–
–	Disagree		22
Locating mental health facilities in a residential area downgrades the neighbourhood	–		–
–	Disagree		19
It is frightening to think of people with mental problems living in residential neighbourhoods	–		–
–	Disagree		19
Having mental patients living within residential neighbourhoods might be good therapy, but risks to residents are too great	–		–
–	Disagree		13

*Notes*: Response options for the MAKS and CAMI included strongly agree, agree, neutral, disagree, strongly disagree and do not know. Possible responses to the RIBS intended behaviour items included agree strongly, agree slightly, neither agree nor disagree, disagree slightly, strongly disagree, do not know. Agree refers to the combined percentage of respondents who answered strongly agree and agree. Disagree refers to the combined percentage of respondents who answered disagree strongly and disagree slightly.
